# Development and Validation of the Pediatric Well-Being Picture Scale© Using a Mixed-Methods Research Design

**DOI:** 10.3390/nursrep15010029

**Published:** 2025-01-20

**Authors:** Judith Quaranta, Rosa Darling, Mei-Hsiu Chen, Julia DeMartino, Madison Kozlowski

**Affiliations:** 1 Decker College of Nursing and Health Sciences, Binghamton University, Binghamton, NY 13902, USA; rdarling@binghamton.edu (R.D.); jdemart4@binghamton.edu (J.D.); mkozlow3@binghamton.edu (M.K.); 2 Department of Mathematics and Statistics, Binghamton University, Binghamton, NY 13902, USA; mchen@binghamton.edu

**Keywords:** child well-being, tool development, mixed-methods research

## Abstract

**Background/Objectives**: Decreased well-being may be a precursor to mental health challenges. Mental health visits for 5–11-year-old children increased by 24% from 2019 to 2020. COVID-19 led to record high levels of anxiety and depression in young children. This highlights the need for early identification and intervention of decreased well-being to prevent progression to potential mental health issues. The purpose of our research was to develop the Pediatric Well-Being Picture Scale© (PWBPS©), the first picture-based screening tool for ages 8–11 years, accessible to children regardless of their literacy, language skill, and developmental levels, allowing for quick screening for early referral and intervention. **Methods**: The mixed-methods research design included focus groups and one-on-one interviews for content and face validity, test/retest reliability, convergent validity, and exploratory factor analysis. Subjects were recruited from public elementary schools. **Results**: The numbers of participating subjects were as follows: N = 17 for focus groups; N = 12 for one-on-one interviews; N = 50 for test/retest reliability; and N = 237 for convergent validity. Thematic analysis resulted in a 10-item, 3-point picture-based Likert scale. The test/retest reliability demonstrated strong correlations, with an ICC of 0.823 (95% CI [0.690, 0.905]). The Cronbach’s alpha for all the administrations was 0.74, 0.74, 0.84, and 0.89. The convergent validity demonstrated correlation with the validated KIDSCREEN-10. The Spearman’s correlation was 0.64 (95% CI as [0.55, 0.71]). The cutoff for the PWBPS© was 18.5, which correlated to a score of 22 on the KIDSCREEN-10. All the items loaded on one component. **Conclusions**: These findings demonstrate that the PWBPS© is valid and reliable, allowing for quick and accurate assessments of children’s well-being and allowing for early intervention, which is key to reducing the negative effects of poor mental well-being.

## 1. Introduction

Well-being allows people to manage their emotions and have a sense of meaning, purpose, and supportive relationships. The World Health Organization (WHO) defines well-being as the ability to realize one’s abilities and be able to cope with life’s normal stressors while working productively and contributing to one’s community [1]. It must be emphasized that well-being is not the same as anxiety or depression. Mental health and mental well-being are different phenomena. Mental ill-health and mental disorders are characterized by abnormal psychological patterns, emotional distress, and impaired functioning [2]. While it has been shown that mental well-being is independently associated with the symptoms of mental illness [3], it is possible for mental well-being to persist even when experiencing mental suffering [2]. Despite moderate negative correlations between anxiety, depression and well-being, they are considered separate measures [4]. While a low level of well-being can lead to depression or anxiety, the relationship is not causal [5]. Burns et al. [6] found that high and moderate levels of well-being were protective against future mental health outcomes.

The need to ensure well-being in our children is imperative in order to address the concerning rise in mental health crises among elementary-aged children. According to the WHO [7], 15% of the global burden of illness and disability in 10–19-year-old individuals is due to mental health disorders such as depression, anxiety, and behavioral disorders. Unfortunately, one in seven of these conditions is untreated due to lack of recognition of the disorder. The Centers for Disease Control and Prevention (CDC) [8] reported that in 2021–2022, 10% of children ages 3–17 had current, diagnosed anxiety (9% of males and 11% of females); 7% of children ages 3–17 had current, diagnosed behavior disorders (10% of males and 5% of females); and 4% of children ages 3–17 had current, diagnosed depression (3% of males and 6% of females). Mass violence, natural disasters, and political polarization, occurring simultaneously with the expected normative developmental emotional crises in this population, have been implicated in placing our youth at a high risk of disruptions to well-being, increasing the risk of mental health disorders in this cohort [9].

The Centers for Disease Control and Prevention [10] reported suicide to be the second leading cause of death in people ages 10–14 in 2022, underpinning the need for early detection of decreased well-being and intervention to remediate this outcome. Confounding this is the COVID-19 pandemic, as the suicide rates among youth ages 5–12 years increased significantly during the pandemic [11]. In 2019, Bitsko et al. [12] analyzed numerous federal data systems that collect data on various indicators of children’s mental health and found that 36.7% of high school students reported persistent feelings of sadness or hopelessness, with 18.8% considering suicide.

These statistics support the need for quick, accurate screening of a child’s well-being, allowing for early intervention to prevent adverse consequences that may arise from unaddressed mental health needs [13]. Through literature searches conducted from September 2019 to the present (using the truncated keywords well-being, child, childhood, assessment, and picture), no well-being screening tools were found similar to the one we developed. The tools located were dependent upon parent or teacher reporting, or were dependent on the child’s reading and comprehension ability. Our novel Pediatric Well-Being Picture Scale© (PWBPS©) uses children’s self-reporting and pictures for each item rather than words, thus removing the barrier that literacy levels can create. This paper will focus on the development and validation of the scale.

### Background and Theoretical Framework

The PWBPS© is based on Guelder’s Well-Being Picture Scale [14]. Dr. Susan Terwilliger began revision of the tool for the pediatric population but never completed the tool or conducted any validity or reliability measures. Our tool development became an extension of Terwilliger’s initial work [15], conducted with her permission.

The foundational underpinning of these tools is Rogers’ [16] science of unitary human beings, which views human beings and the environment as energy fields. Emphasis is placed on the coexistence, and reciprocal relationship, of the human and their environment and its influence on health. The human–environment field is a dynamic, open system in which change is continuous and innovative. This constant interchange results in a system that is never the same at any two moments; it is continually new or different. Rogers viewed the individual holistically, in an integral and intertwined relationship with the environment, which predicts consequential changes in the life process. Continuous changes in human behavior and life processes occur as a result of the simultaneous interaction of the actual state of the human field and the environmental field’s actual state at any given point [17].

Gueldner et al. [14] postulated that these changes that occur from the human–environment interaction are associated with a sense of well-being. The picture scales of both Gueldner and Terwilliger were designed to assess the human–environmental energy field: (a) frequency of movement (intensity) within the energy field, (b) awareness of one’s self as energy, (c) action emanating from the energy field, and (d) power within the mutual human–environmental energy field process. Terwilliger et al. [15] suggested that individuals experience well-being during times of higher frequency and harmony within their mutual human–environmental field process. Based on the research by Gueldner [14] and Terwilliger [15], the picture scale items of the PWBPS© that assess frequency include the eye, candle, and butterfly; awareness is assessed by the pencil, puzzle, faces, and sun items; action is assessed by the man and children; and power is assessed by the lion item. Since Terwilliger’s scale was not a validated tool, further development and testing were vital to create a meaningful screening tool for well-being.

## 2. Materials and Methods

Human subjects approval from Binghamton University was obtained for all the phases of development and validity and reliability testing for the PWBPS© (focus groups: STUDY00002180 approved on 7 February 2020; test/retest: STUDY 00004101 approved on 21 February 2023; convergent forms: study 00004101 approved on 21 February 2023). Parental informed consent and child informed assent were obtained prior to each phase of the study. Intellectus Statistics [18], an online computer software program, was used to analyze data imported from IBM SPSS Statistics, version 28. An a priori alpha level of 0.05 was used for all the quantitative analyses. This study was conducted in three phases: focus group/interview, test/retest, and convergent validity. Tool completion took 4 years. The inclusion criteria for all the phases consisted of children ages 8–11 years or in grades 3–5, and able to read and speak English.

### 2.1. Focus Group and One-on-One Interviews for Tool Development

The exclusion criteria consisted of a lack of computer access. An email invitation was sent to the parents of all children in grades 3–5 in a local school district, which included a description of the study and a Qualtrics link to provide parental consent electronically. Assent was obtained from the children whose parents consented through a private Zoom session prior to conducting the focus groups. The focus groups were conducted virtually through Zoom, with two to three researchers facilitating each focus group. All the focus groups were recorded.

Fidelity and trustworthiness were addressed. Fidelity, ensuring that all the participants in a study receive the same conditions [19], was achieved through training the researchers in focus group facilitation. All the researchers participated in the development and implementation of the script and focus group questions. Trustworthiness, essential to ensure confidence in the findings, includes credibility, transferability, and dependability [20]. Credibility occurs when multiple researchers complete comparative analyses of individual findings, and when participants verify the researchers’ interpretation of the findings [21]. For the development of the PWBPS©, all the researchers individually analyzed the transcriptions from all the focus groups, then met as a group to reach to a consensus regarding the themes from the focus groups. These findings were then validated with the focus group participants. Transferability expands understanding of findings by comparing them to similar findings from other contexts [21]. This was accomplished through the analysis of published manuscripts related to Gueldner’s and Terwilliger’s previous well-being tools. Dependability, or peer debriefing, involves reading and reacting to another researcher’s notes and interpretations [21], which was accomplished as part of the thematic analysis. Two researchers independently transcribed and analyzed their own recording for the focus group they facilitated. These two researchers then met and discussed their findings. After this was completed for all the focus groups, the entire group of researchers discussed their findings and achieved a consensus.

**Figure 1:**
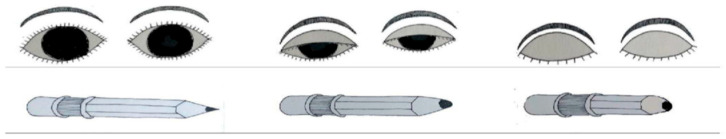
Sample PWBPS© items.

This phase of the study consisted of one focus group session and one one-on-one interview. The focus group session consisted of looking at the existing tool developed by Terwilliger, with two main questions: (a) When you see this picture, what feeling do you think of? and (b) Is there a better picture that shows that feeling? Thematic analysis [22] was used to identify, analyze, organize, describe, and report the themes from the focus group transcriptions. The analysis involved six steps: familiarization, coding, generating themes, reviewing themes, defining and naming themes, and producing the report. All the researchers were trained in this analysis and contributed to all the steps. Once the final tool was created, one-on-one interviews were held where the children reviewed the tool and provided feedback to ensure the tool had captured what they had conveyed in the focus group. All the children who participated in both sessions received a USD 10 gift card. The resulting tool consisted of 10 Likert-style items with three pictures each that represented a continuum of the variables included in the tool. See Figure 1 for sample scale items.

### 2.2. Test/Retest Reliability Testing

Test/retest reliability is assessed by administering the tool to the same people on different occasions and calculating the correlation between the scores obtained [23]. We administered the tool three times for our reliability test. The majority of the children completed the tool three times on separate days within one week. For those children unavailable for the third administration, the time frame between the second and third administration averaged two weeks. Due to the difficulty of recruiting, we administered the tool either in person or virtually through Zoom. All three attempts for each child were performed in the same manner. Subjects were recruited from a local school district (in-person testing) and from a digital newsletter for a local university (virtual testing). Subjects from an additional school district were also recruited virtually. All the parents were provided with a Qualtrics link that explained the study and provided a consent form to be completed electronically. All the children provided assent prior to completing the tool the first time. Only those children who completed the three attempts were included in the analysis. All the children who completed the tool two or three times received a USD 10 gift card.

An iPad was used to administer the tool during the in-person sessions. Demographic information was collected. To control for reading level and comprehension, all the directions for tool completion were provided via audio from the iPad. Each item contained three pictures that were presented at the same time. The child only saw one item at a time. The prompt for each item stated “Please pick the picture that best describes how you feel today”. The child then hit the arrow at the bottom of the screen to advance to the next item. If the child did not select an item, they heard the prompt “You did not select a picture. Are you sure you want to go the next screen?” The child was not required to choose a picture before advancing to the next screen. At the conclusion of the last item, the child was thanked for their participation. Their data were saved on a secure, password-protected server.

For those children who participated in the virtual Zoom format, the same prompts were used but were verbalized by the researcher. The child saw each three-picture item one screen at a time. The researcher recorded their answers, which were then saved on a secure, password-protected server.

The test/retest reliability for the PWBPS© was assessed using a two-way mixed-methods effects model, calculating the intraclass correlation coefficient (ICC) using absolute agreement. Based on the 95% confidence interval of the ICC estimate, values less than 0.5 are indicative of poor reliability; between 0.5 and 0.75 are indicative of moderate reliability; between 0.75 and 0.9 are indicative of good reliability; and greater than 0.90 are indicative of excellent reliability [24].

### 2.3. Convergent Validity

Convergent validity, a subset of construct validity, measures the correlation between two tests that are designed to measure the same thing. The convergent validity is generally considered adequate if a correlation with an instrument measuring the same construct is >0.50 [25].

The KIDSCREEN-10 (KS 10) index child and adolescent questionnaire, which contains 10 items to measure general health-related quality of life, was used for this validity testing. In addition to the good internal consistency (Cronbach’s alpha = 0.82), the good test/retest reliability (r = 0.73; ICC = 0.72) indicates that the index is a precise and stable measurement instrument for health-related quality of life [26]. Permission was obtained to use the tool at the time of implementation, but it should be noted that this tool is now open access.

All the students in grades 3–5 in a local school district were invited to participate. Parental consent was passive. An email was sent by the school district with a Qualtrics link to the study information and the opportunity to opt out. The child assent form, KS 10, and PWBPS© were distributed in the children’s classrooms. To ensure the confidentiality of those who participated in this study, the children were instructed not to answer the questions if they did not want to participate, but to write their name on the first page and turn the packet in with the rest of the class. Children whose parents opted out were provided with the tool, but their responses were not included in the analysis.

### 2.4. Cronbach’s Alpha Coefficient

A Cronbach’s alpha coefficient was calculated each time the tool was administered. The Cronbach’s alpha coefficient was evaluated using the guidelines suggested by Pallant [23] (p. 104), where values above 0.7 are acceptable and values above 0.8 are preferred.

## 3. Results

The PWBPS© was administered a total of four times during the course of this study. The child was able to complete each item within 30 s, accounting for tool completion of the 10 items in less than five minutes. Each item was given a value of 1, 2, or 3. Four items were reverse scored. All the children whose parents gave consent assented to participate in this study.

### 3.1. Demographic Information

Demographic information for each phase of the project can be found in Table 1. It should be noted that the most represented age group is 9 years old. Gender was self-identified by the participants. Due to the low N for some of the age groups, the gender distribution by age group was not included to reduce the risk of violating the confidentiality of the participants. Due to the lack of racial diversity of the study location, race was also not included to reduce the risk of violating confidentiality. An independent-samples t-test was conducted to compare gender and scores on the PWBPS©. A significant difference was found, with males (*M* = 24.1, *SD* = 4.56) scoring higher than females (*M* = 22.26, *SD* = 5.13); *t*(260) = 2.95. *p* = 0.03. A one-way between-groups analysis of variance was conducted to explore the impact of age on scores on the PWBPS©. There was a statistically significant difference between the age groups at the *p* < 0.05 level: *F*(3, 255) = 3.26, *p* = 0.022. Post hoc comparisons using the Bonferroni correction indicated that the mean score for 8 year olds (*M* = 23.94, *SD* = 5.04) was significantly different than that for 11 year olds (*M* = 21.05, *SD* = 4.77). The mean score for 9 year olds (*M* = 23.54, *SD* = 5.05) was significantly different than that for 11 year olds (*M* = 21.05, *SD* = 4.77).

### 3.2. Validity Testing via Focus Groups

A total of 25 parents provided consent for their children. Seventeen children provided assent and participated in the focus groups. Each focus group had between three and five participants. Of the 17 focus group participants, 12 participated in the follow-up one-on-one interview sessions, resulting in a 70% completion rate.

Revisions were made to the tool based on the first focus group discussions. Based on the transcribed responses of the focus group participants, each item of the scale was evaluated and revised to reflect the feelings and emotions elicited when viewing the pictures. All the children felt that a four-item scale was too difficult and recommended that a three-item scale be adopted. Some of the pictures from the original tools were deleted. A local artist revised the pictures.

The one-on-one interviews with the children provided the face and content validity of the PWBPS©. During these interviews, the participants were shown the revised tool. Due to the timing of the one-on-one interviews, which occurred over summer 2021, the focus group setting was not feasible. Each child was asked if they felt the tool reflected their suggestions. They were then asked to identify what feeling they associated with each item of the tool. See Table 2 for the consensus on the meaning of each item of the PWBPS©.

### 3.3. Test/Retest Reliability

A total of 54 children participated in this phase of the tool development. Thirty-five completed the tool three times, and an additional fifteen completed the tool two times. The test/retest reliability of the measure, as assessed by the ICC (two-way mixed effects, absolute agreement), was found to be 0.823 (95% CI [0.690, 0.905]).

### 3.4. Convergent Validity

**Figure 2:**
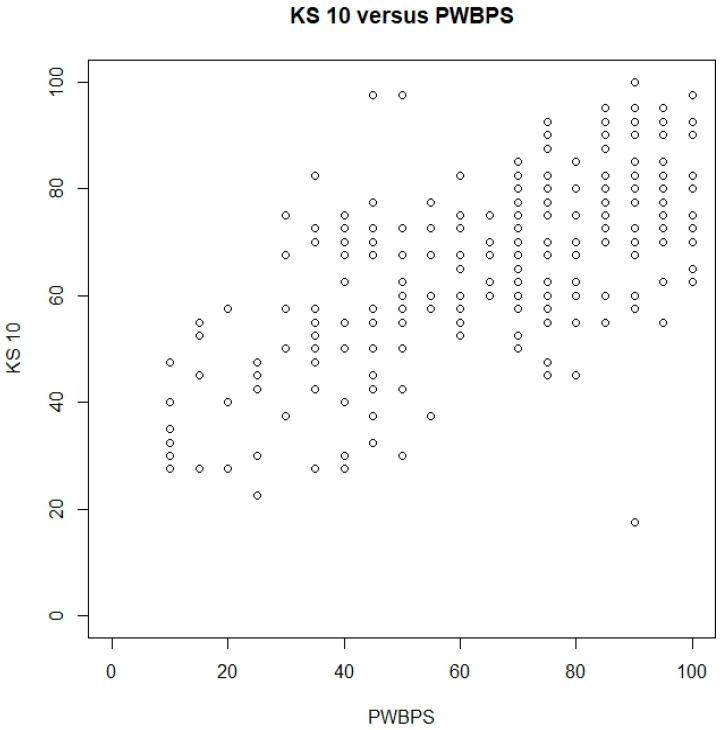
Scatterplots for the KS 10 and PWBPS©.

**Figure 3:**
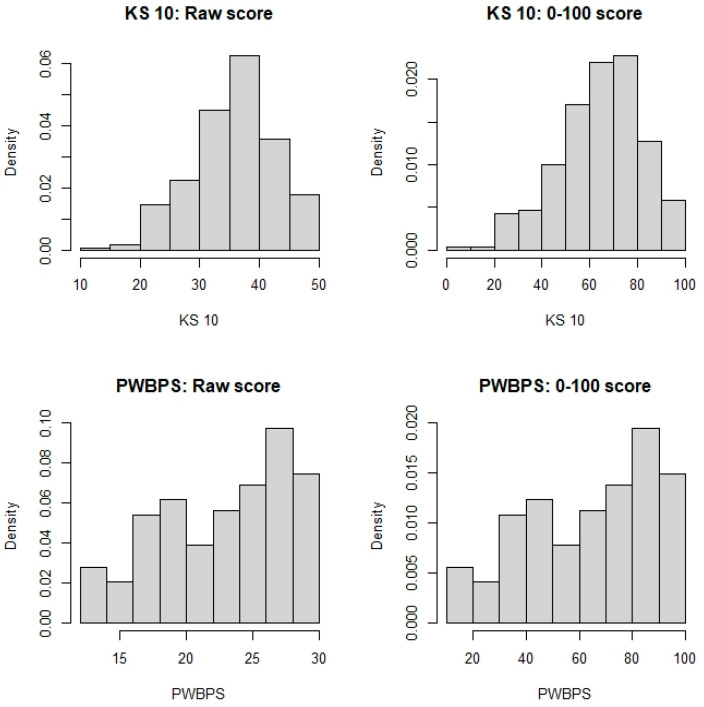
Conversion of the KS 10 and PWBPS© raw scores to 0–100 scores.

**Figure 4:**
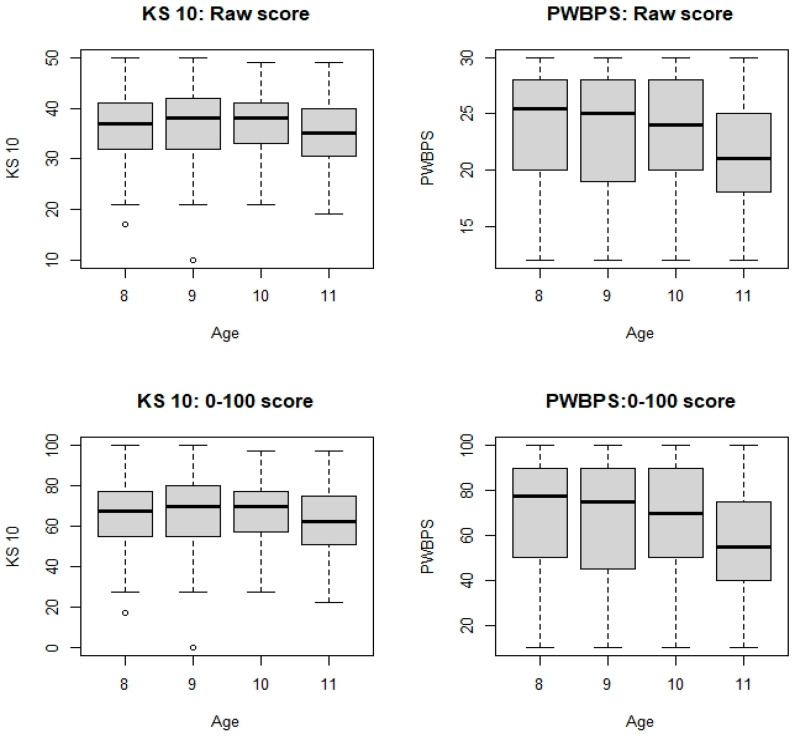
Difference in scores by age.

**Figure 5:**
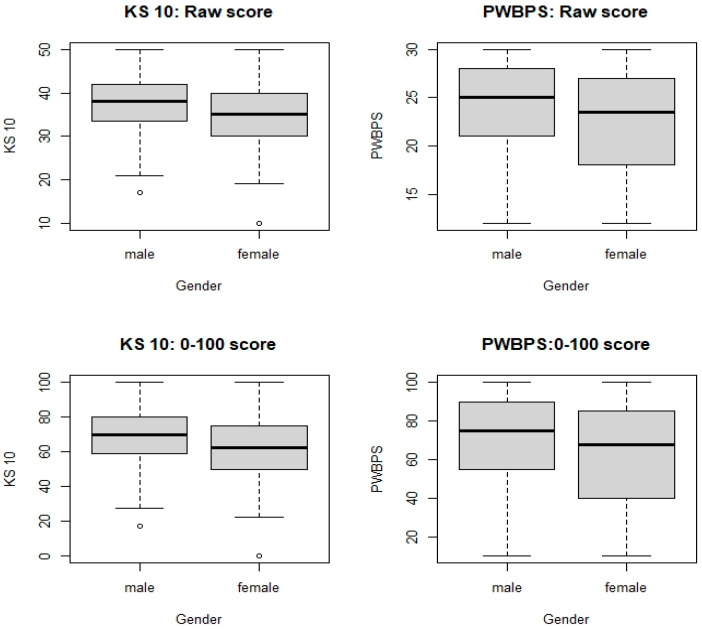
Difference in scores by gender.

**Figure 6:**
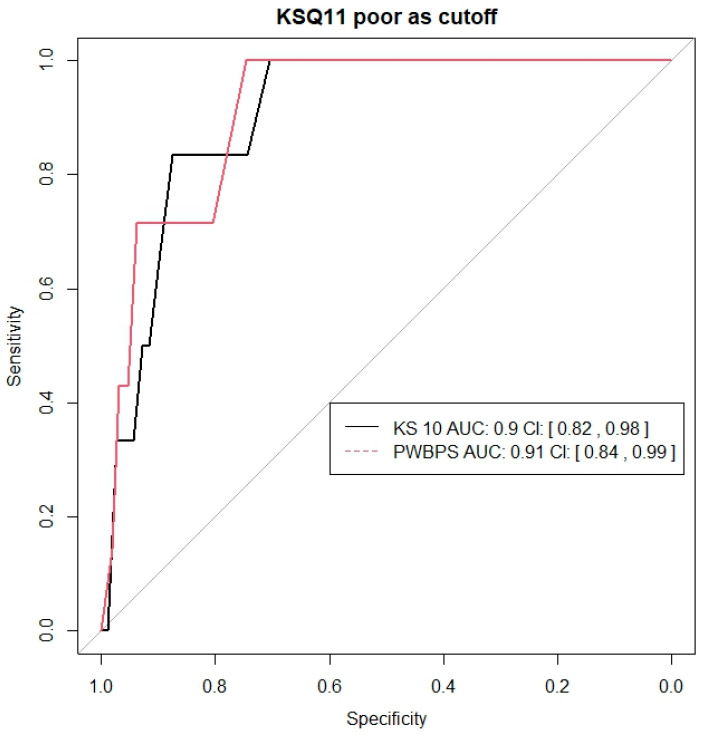
Receiver operating characteristic curve analysis based on the summary response of “Poor” versus others from the KS 10.

A total of 297 children ages 8–11 participated in this part of the present study. See Table 1 for demographic information. A total of 237 children completed all the items of both the KS 10, with scores ranging from 10 to 50, and the PWBPS©, with scores ranging from 10 to 30. The Spearman’s correlation was estimated to be 0.64 (95% CI [0.55, 0.71], indicating that as the KS 10 scores become higher, the PWBPS© also tends to score higher. Furthermore, when converting to 0–100 scores, the two tools’ centers are similar, but with PWBPS© having more variation (see Figure 2). Figure 3 demonstrates these similarities and Table 3 shows the scores by quartile for each instrument. For the KS 10 T-values, the sample mean and median were around 45. For both tools, there were noted age and gender effects. While the KS 10 does not seem to vary by age, the PWBPS© scores tend to decrease as the age increases. See Figure 4 for box plots that demonstrate the scores by age. Additionally, females tended to score lower compared to males. See Figure 5 for box plots that demonstrate the gender differences. Based on the ruler of the response from the KS 10 manual, with a cutoff for the KS 10 T-value = 30 (or 22 in the original score or 30 in the 0–100 score) as a reference, the area (AUC) under the receiver operating characteristic (ROC) curve of the PWBPS© was estimated to be 0.86 (95% confidence interval [0.73, 0.99]), with an estimated cutoff of 18.5 in the original score (or 42.5 in the 0–100 score) for the PWBPS©. Using the summary question from the KS 10, the AUCs using the PWBPS© and KS 10 in predicting a “poor” response were estimated to be 0.91 and 0.90, respectively (Figure 6). Based on the ROC analysis, the cutoff score where a referral is recommended to be initiated to further assess the child was estimated to be 15.5 in the original score (or 27.5 in 0–100 score) for the PWBPS© and a cutoff value of 28.5 in the original score (or 46.25 in the 0–100 score) for the KS 10.

### 3.5. Exploratory Factor Analysis

A principal component analysis (PCA) was run on the 10-question well-being scale. The suitability of the PCA was assessed prior to the analysis. Inspection of the correlation matrix showed that all the variables had at least one correlation coefficient greater than 0.3. The overall Kaiser–Meyer–Olkin (KMO) measure was 0.92, with individual KMO measures all being greater than 0.8, which are classifications of “meritorious” to “marvelous” according to Kaiser [27]. Bartlett’s test of sphericity was statistically significant (*p* < 0.001), indicating that the data were likely factorizable.

The PCA revealed one component that had an eigenvalue greater than one and which explained 51.6% of the total variance. Visual inspection of the scree plot indicated that only one component should be retained [28]. The interpretation of the data was consistent with the well-being factors the questionnaire was designed to measure, with strong loadings in the factors measuring awareness of one’s self as energy components, action emanating from the energy field component, and power with the mutual human–environmental energy field process component. One item was less correlated with the others in the frequency of movement with the energy field component. We chose to keep this item in the scale since it measures an important construct and its removal did not change the reliability of the scale. Consideration of removal of this item is an area for future research with a larger and more diverse sample of participants. 

### 3.6. Cronbach’s Alpha Coefficient

A Cronbach’s alpha coefficient was calculated each time the PWBPS© was administered. The items for the first administration for the test/retest (N = 50) had a Cronbach’s alpha coefficient of 0.74, indicating acceptable reliability. The items for the second administration for the test/retest (N = 50) had a Cronbach’s alpha coefficient of 0.74, indicating acceptable reliability. The items for the third administration for the test/retest (N = 50) had a Cronbach’s alpha coefficient of 0.84, indicating good reliability. The fourth administration for convergent validity had a Cronbach’s alpha coefficient of 0.89, indicating good reliability (N = 235).

## 4. Discussion

The development of the PWBPS© was centered around school-aged children ages 8–11. Considering the amount of time children spend at school and the importance it plays in their growth and development, it is perhaps one of the most ideal locations to screen children utilizing the PWBPS©.

Bitsko et al. [12] analyzed mental health data from multiple agencies from 2013 to 2019 and found that mental health disorders begin in early childhood. Their findings show that almost 10% of children ages 3–17 were affected by anxiety and 20% of children ages 12–17 experienced a major depressive episode. Feelings of sadness or hopelessness were reported by 36.7% of high school students, with 18.8% considering suicide. These statistics illustrate the need for a quick and effective screening tool that identifies children at risk and allows for early intervention.

The goal throughout the research, modification, and testing of this tool was to provide a quick and effective way to screen the pediatric population to identify those children whose well-being may be at risk. Completing face and content validity testing in focus groups and one-on-one interviews ensured that the tool captured the feelings and emotions that children experience in their daily lives.

The results concerning the test/retest reliability demonstrated strong correlations among the three separate administrations of the PWBPS©, supporting the finding that the PWBPS© measures well-being consistently over time. The convergent validity, assessed by comparing the PWBPS© to the KS 10, also supported the validity of the PWBPS© as a well-being screening tool for children ages 8–11. While not intended as a diagnostic tool, the PWBPS© does allow school personnel or healthcare providers to screen children within five minutes to see how they are feeling on a given day. Scores below 18.5 on the PWBPS© warrant further conversation to see what is happening in that child’s life. The results from the exploratory factor analysis and Cronbach’s alpha also supported scale items that consistently measure well-being, indicating that the PWBPS© is a valid and reliable screening tool for children ages 8–11.

### 4.1. Relevance to Nursing Practice

Prior to the development of this tool, the well-being of a child was dependent upon guardian-reported assessments or the ability of the child to comprehend a written assessment. These were barriers to the accurate assessment and reporting of well-being. The PWBPS© overcomes these barriers as the child selects pictures associated with how they feel, providing a more accurate depiction of well-being through self-reporting. As such, one of the main beneficial components of the PWBPS© is that it does not have a language component, and therefore, can be taken by any child regardless of their language or literacy level. Diverse populations that speak a variety of languages and come from different ethnic backgrounds provide a challenge when a screening tool needs to be accurately translated and validated in different languages. 

Mental health screening and early intervention are vital in terms of identifying and supporting students who may be struggling with psychological or psychosocial challenges. The PWBPS© is a tool for early detection of low overall well-being that identifies children who may be at risk, allowing for timely interventions that can prevent academic, social, and emotional difficulties from escalating.

### 4.2. Limitations

The recruitment of subjects from the local schools was challenging. The schools were sometimes hesitant to add another activity to an already packed schedule. All the participation that depended on parental consent was low. It was difficult to get parents to respond to the email invitations.

The PWBPS© is currently only validated for children 8–11 years of age. We cannot guarantee that a cutoff score of 18.5 is accurate for children outside this age range. Our sample was primarily White due to the location in which the tool was administered. Future research will include a more heterogenous sample and testing that includes ages older than 11 and younger than 8 to increase the generalizability of this tool.

## Figures and Tables

**Table 1:** Demographic information for each phase of the tool development.

Method	N	Age	Gender
Focus Group (RR = 21%)	17	8 years: 5	Male: 41% Female: 59%
		9 years: 10	
		10 years: 1	
		11 years: 1	
One-on-One Interview (RR = 71%)	12	8 years: 2	Male: 25% Female: 75%
		9 years: 8	
		10 years: 1	
		11 years: 1	
Test/Retest (RR = 15%)	50	8 years: 13	Male: 56% Female: 44%
		9 years: 17	
		10 years: 17	
		11 years: 3	
Convergent Validity (RR = 86%)	297	8 years: 56	Male: 50% Female: 48%
		9 years: 104	
		10 years: 85	
		11 years: 44	
		No response: 8	

Note. RR = response rate.

**Table 2:** Consensus on the meaning of each item of the Pediatric Well-Being Picture Scale©.

Picture	Theme	# Consensus	%
Eyes	Wakefulness	12	100
Faces	Happiness	12	100
Lion	Growing	10	83
Candle	Burned Out	12	100
Sun	Emotions	11	92
Man	Energy	8	67
Puzzle Pieces	Completeness	11	92
Butterfly	Change	10	83
Kids	Being Included	11	92
Pencil	Hard Work	9	75

Note. N = 12.

**Table 3:** Summary of the raw and 0–100 scores for the KS 10 and PWBPS© by quartile.

Scale	Minimum	1st Quartile	Median	Mean	3rd Quartile	Maximum
KS 10 raw score	10	32	37	36.24	41	50
KS 0–100 score	0	55	67.5	65.6	77.5	100
PWBPS© raw score	12	19	24	23.27	28	30
PWBPS© 0–100 score	10	45	70	66.36	90	100

## Data Availability

Data are available upon request from the corresponding author due to the privacy of participants.

## Public Involvement Statement

The participants in these studies were children ages 8–11. Their participation is explained in the Section 2 of the article.

## Guidelines and Standards Statement

This manuscript was drafted in accordance with the observational studies guidelines (Equator Network. (n.d.). Enhancing the QUALity and transparency of health research. Retrieved 26 October 2024 at https://www.equator-network.org/).
